# Short forms of the Child Perceptions Questionnaire for 11–14-year-old children (CPQ_11–14_): Development and initial evaluation

**DOI:** 10.1186/1477-7525-4-4

**Published:** 2006-01-19

**Authors:** Aleksandra Jokovic, David Locker, Gordan Guyatt

**Affiliations:** 1 Community Dental Health Services Research Unit, Faculty of Dentistry, University of Toronto, 124 Edward Street, Toronto, Ontario, M5G 1G6, Canada; 2 Department of Clinical Epidemiology and Biostatistics, McMaster University, Hamilton, Ontario, Canada

## Abstract

**Background:**

The Child Perceptions Questionnaire for children aged 11 to 14 years (CPQ_11–14_) is a 37-item measure of oral-health-related quality of life (OHRQoL) encompassing four domains: oral symptoms, functional limitations, emotional and social well-being. To facilitate its use in clinical settings and population-based health surveys, it was shortened to 16 and 8 items. Item impact and stepwise regression methods were used to produce each version. This paper describes the developmental process, compares the discriminative properties of the resulting four short-forms and evaluates their precision relative to the original CPQ_11–14_.

**Methods:**

The item impact method used data from the CPQ_11–14 _item reduction study to select the questions with the highest impact scores in each domain. The regression method, where the dependent variable was the overall CPQ_11–14 _score and the independent variables its individual questions, was applied to the data collected in the validity study for the CPQ_11–14_. The measurement properties (i.e. criterion validity, construct validity, internal consistency reliability and test-retest reliability) of all 4 short-forms were evaluated using the data from the validity and reliability studies for the CPQ_11–14_.

**Results:**

All short forms detected substantial variability in children's OHRQoL. The mean scores on the two 16-item questionnaires were almost identical, while on the two 8-item questionnaires they differed by only one score point. The mean scores standardized to 0–100 were higher on the short forms than the original CPQ_11–14 _(p < 0.001). There were strong significant correlations between all short-form scores and CPQ_11–14 _scores (0.87–0.98; p < 0.001). Hypotheses concerning construct validity were confirmed: the short-forms' scores were highest in the oro-facial, lower in the orthodontic and lowest in the paediatric dentistry group; all short-form questionnaires were positively correlated with the ratings of oral health and overall well-being, with the correlation coefficient being higher for the latter. The relative validity coefficients were 0.85 to 1.18. Cronbach's alpha and intraclass correlation coefficients ranged 0.71–0.83 and 0.71–0.77, respectively.

**Conclusion:**

All short forms demonstrated excellent criterion validity and good construct validity. The reliability coefficients exceeded standards for group-level comparisons. However, these are preliminary findings based on the convenience sampling and further testing in replicated studies involving clinical and general samples of children in various settings is necessary to establish measurement sensitivity and discriminative properties of these questionnaires.

## Background

Measures of oral-health-related quality of life (OHRQoL) provide essential information when assessing the treatment needs of individuals and populations, making clinical decisions and evaluating interventions, services and programs. The only measures of this kind currently available for children are the Child Oral Health Quality of Life (COHQoL) questionnaire [1-4] and the Child-Oral Impacts on Daily Performances (Child-OIDP) [5].

The COHQoL is a set of multidimensional scales measuring the negative effects that oral and oro-facial diseases and disorders may have on the well-being of 6–14-year-olds and their families. One of its components is the Child Perceptions Questionnaire for children aged 11 to 14 years (CPQ_11–14_) [1].

The CPQ_11–14 _consists of 37 questions organized into four health domains: oral symptoms (n = 6), functional limitations (n = 9), emotional well-being (n = 9) and social well-being (n = 13). The questions ask about the frequency of events in the previous three months in relation to the child's oral/oro-facial condition. The response options are: 'Never' = 0; 'Once/twice' = 1; 'Sometimes' = 2; 'Often' = 3; 'Everyday/almost everyday' = 4. The questionnaire also contains global ratings of the child's oral health and the extent to which the oral/oro-facial condition affected his/her overall well-being. They are worded as follows: "Would you say that the health of your teeth, lips, jaws and mouth is..." and "How much does the condition of your teeth, lips, jaws or mouth affect your life overall?" A 5-point response format ranging from 'Excellent' = 0 to 'Poor' = 4 and from 'Not at all' = 0 to 'Very much' = 4, respectively, is offered for these ratings.

**Figure 1:**
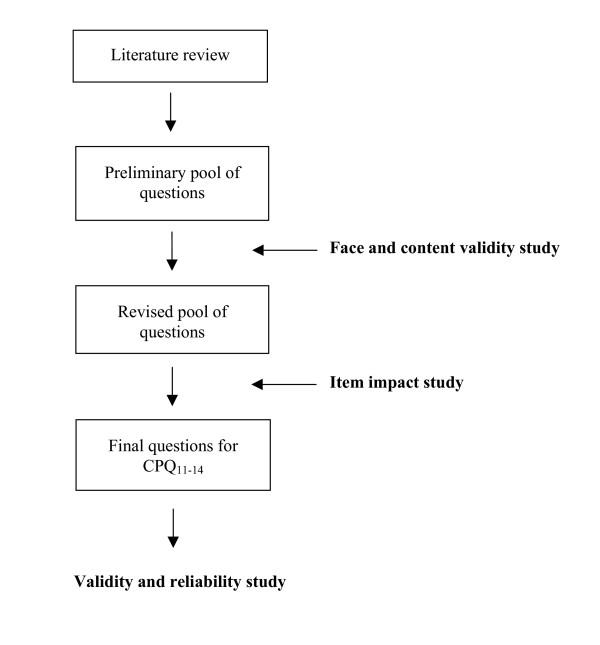
Development of the long-form CPQ_11–14 _Questions.

The CPQ_11–14 _was constructed using a systematic multistage process based on the theory of measurement and scale development [6,7]. The process for the development and evaluation of health-related quality of life (HRQoL) measures described by Guyatt et al. [8] and Juniper et al. [9] was followed (Figure 1). The defining characteristic of the development process used is the item impact study, which selects questions for a final questionnaire from an initial pool of questions based on their impact scores. Impact scores are obtained by multiplying the frequency of the experience addressed by each question and the mean rating of the emotional response it evokes in the children studied. A detailed description can be found in other publications [1-4]. Participants in both the development and evaluation of the CPQ_11–14 _were children with dental caries (paediatric dentistry group), malocclusions (orthodontic group) and clefts of the lip and/or palate (oro-facial group). The recruitment process and sample characteristics have also previously been published [1].

The CPQ_11–14 _performed well as a discriminative measure, being able to distinguish between the three groups, and showed excellent internal consistency (α = 0.91) and test-retest reliability (ICC = 0.90) [1]. Cronbach's alphas for the four domains ranged from 0.64 to 0.86 and ICCs from 0.79 to 0.88. Nevertheless, the use of the measure in clinical settings and large scale population surveys may be limited by its length and the burden placed on respondents. A short form would broaden its applications, by reducing the time and financial costs of data collection and the risk of total and item non-response.

Although short forms of many commonly used instruments have been developed no guidelines have been published with respect to the methods that should be used to select items for a short form [10]. Coste et al [10] reviewed 42 studies in which medical, psychological or educational measures had been shortened and found that most aimed to produce a form that was easier and more practical to use rather than a form that had enhanced psychometric properties. The most common approach to producing a short form was statistical with factor analysis, correlation and stepwise regression analysis being the favoured techniques for selecting items. Expert opinion alone or in combination with these statistical techniques was also used. Although statistical approaches are well-established [11-14], Coste et al [10] consider most to be inappropriate in the majority of cases.

Juniper et al [11] recommend the use of item impact methods whereby items are selected that are deemed to be the most important by patients. They compared the use of the item impact method and factor analysis when shortening the Asthma Quality of life Questionnaire. The two approaches resulted in very different instruments. The former produced a 32-item instrument and the latter 36-item measure, with only 20 items being common to both. Factor analysis resulted in the deletion of several items of importance to patients with asthma. However, they did not compare the psychometric properties of the two short-forms.

Locker et al [15] compared the content and properties of two 14-item versions of the Oral Health Impact Profile [13], a 49-item measure of the quality of life outcomes of oral disorders for use in older adult populations. One version was developed using a stepwise regression approach and the other using an item impact approach. The short forms had only two items in common. Because of its content, the regression short form was better at discriminating between groups but had marked floor effects. The impact short form had minimal floor effects and was more sensitive to change.

Based on the results of these studies we decided that there was a sound philosophical and methodological rationale for the use of the item impact approach to develop a short form of the CPQ_11–14_. Since this approach is only feasible if an item impact study has been undertaken, we also used a stepwise regression approach that can be applied to any data set in which the measure of interest has been used, with the intention of comparing the two methods. The regression approach was chosen over other statistical methods because it had been used previously in shortening oral health-related quality of life questionnaires [13].

No guidelines concerning how short a short-form should be have been published. Four items per domain is considered a minimum number of questions that is required to control for random error (i.e. to minimize the effect of idiosyncratic responses to the individual questions) and to allow within-domain analysis [11]. Consequently, we aimed to develop a 16-item version of the CPQ_11–14 _with four items in each of the four domains. In order to determine if the properties of a measure can be maintained when a substantial proportion of the items are deleted, we also developed an 8-item measure, with two items per domain, even though a measure of this length would not be suitable for within-domain analysis. The versions developed using the item impact method are referred to as the CPQ_11–14_-ISF:16 and the CPQ_11–14_-ISF:8. The CPQ_11–14_-RSF:16 and CPQ_11–14_-RSF:8 denote the versions developed using the regression method.

This paper describes the development of the short forms and compares the content and properties (i.e. cross-sectional validity and reliability) of the 16 and 8-item versions derived using the two methods. It also describes the performance of the short-form questionnaires relative to the original CPQ_11–14 _in terms of the measurement sensitivity and precision. The latter involved comparisons of the reliabilities and assessments of the relative validity of the short-forms.

## Methods

### Development

**The item impact method **of developing short forms used the data obtained during the CPQ_11–14 _item impact study. Here, children (n = 83) from the three clinical groups defined above participated in face-to-face interviews using a form consisting of questions from the preliminary item pool (Figure 1). The children were asked whether they experienced the problem described by each question and, if yes, indicated its importance on a 4-point scale ranging from 0 ("Does not bother me at all") to 4 ("Bothers me very much"). The questions were then ranked within health domains according to their impact scores, which represent products of the question frequency and the mean bother rating. The top 4- and 2-ranked questions in each domain were selected for the CPQ_11–14_-ISF:16 and the CPQ_11–14_-ISF:8, respectively (Table 1 &2).

**Table 1:** Questions in the CPQ_11–14 _– ISF :16 and the CPQ_11–14 _– RSF:16

**In the past 3 months, how often have you ... (had/been) ... because of your teeth/mouth?**			
**Domain**	**ISF specific questions**	**Common questions**	**RSF specific questions**

**OS**^**a**^		Pain in teeth/mouth Bad breath Mouth sores Food caught between teeth	
**FL**^**b**^	Difficulty eating/drinking hot/cold foods	Difficulty chewing firm foods Difficulty saying words Taken longer to eat a meal	Trouble sleeping
**EW**^**c**^		Upset Felt irritable/frustrated Felt shy Concerned what people think about your teeth/mouth	
**SW**^**d**^	Asked questions	Teased/called names Avoided smiling/laughing Argued with children/family	Not wanted to speak/read loud in class

^a ^Oral Symptoms, ^b ^Functional Limitations, ^c ^Emotional well-being, ^d ^Social well-being

**Table 2:** Questions in the CPQ_11–14 _– ISF :8 and the CPQ_11–14 _– RSF:8

**In the past 3 months, how often have you ... (had/been) ... because of your teeth/mouth?**			
**Domain**	**ISF specific questions**	**Common questions**	**RSF specific questions**

OS^a^	Food caught between teeth	Bad breath	Mouth sores
FL^b^	Difficulty chewing firm foods Difficulty eating/drinking hot/cold foods		Difficulty saying words Trouble sleeping
EW^c^	Felt irritable/frustrated	Upset	Concerned what people think about your teeth/mouth
SW^d^	Avoided smiling/laughing Asked questions		Teased/called names Argued with children/family

^a ^Oral Symptoms, ^b ^Functional Limitations, ^c ^Emotional well-being, ^d ^Social well-being

**The regression method **was applied to the data collected in the study that evaluated the validity of the CPQ_11–14 _(n = 123). The dependent variable was the overall score for the long-form CPQ_11–14 _calculated by summing the response codes to its 37 questions. The independent variables were the scores for individual questions in the CPQ_11–14_. A single model was generated with all items included and a forward stepwise procedure used to identify the best predictors of the overall score. The 4 and 2 questions from each health domain entering the model and making the largest contribution to the coefficient of variation (R^2^) were selected for the CPQ_11–14_-RSF:16 and the CPQ_11–14_-RSF:8, respectively (Table 1 &2).

### Evaluation

The measurement properties of the CPQ_11–14_-ISF-16; the CPQ_11–14_-ISF-8; the CPQ_11–14_-RSF-16 and the CPQ_11–14_-RSF-8 were evaluated using the data from the validity and reliability studies for the long-form CPQ_11–14 _[1]. Scores for all short forms were calculated by summing the response codes to their questions. Criterion validity, construct validity and internal consistency reliability were assessed based on the responses from 123 children. Clinical data were obtained for 26 of the paediatric dentistry group, 45 of the group with malocclusions and all 39 of the oro-facial group and used for further assessments of construct validity. Sixty-five of the 123 children, who completed the CPQ_11–14 _again after a period of two weeks and who did not report change in either their oral health or its impact on their overall well-being at the follow-up, provided the data for the assessment of test-retest reliability.

For criterion validity, positive high correlations between the long-form and each short-form questionnaire were expected. For discriminant construct validity, the hypothesis that the scores are highest in the oro-facial, lower in the orthodontic and lowest in the paediatric dentistry group was tested. It was also hypothesized that within each of the three groups scores would be highest for those with the most severe clinical condition. For correlational construct validity, positive correlations between the scores and children's global ratings of oral health and well-being were tested. Since the former is a measure of health and the latter a measure of health-related quality of life, it was predicted that the correlation coefficient would be higher for the rating of well-being than for the rating of oral health.

Relative validity (RV) estimates were computed as the ratios of F statistics for the short-form questionnaires and the original CPQ_11–14_. They indicate in proportional terms how much more or less precise a short-form questionnaire is in relation to the original CPQ_11–14 _[16,17].

Internal consistency reliability was determined determined using Cronbach's alpha. Alphas were also calculated with each item deleted. Corrected item total correlations were also compared. Test-retest reliability was assessed using the intraclass correlation coefficient (ICC). This was calculated using a one-way analysis of variance random effects parallel model [18,19].

## Results

### Content of the questionnaires

As Table 1 &2 show, the CPQ_11–14_-ISF:16 and CPQ_11–14_-RSF:16 are very similar as they share 14 of their 16 items. The questions specific for the CPQ_11–14_-ISF:16 concern temperature sensitivity and being asked about the condition of teeth/mouth, while those specific for the CPQ_11–14_-RSF:16 concern trouble sleeping and not wanting to speak in class. On the contrary, the CPQ_11–14_-ISF:8 and the and CPQ_11–14_-RSF:8 have only 2 questions in common: 'Bad breath' and 'Been upset'.

### Descriptive statistics

The scores indicated that all short-forms detected substantial variability in children's perceptions of their OHRQoL (Table 3). Floor-effects were almost non-existent, with only 0.8% and 4.1% of children having zero scores on the CPQ_11–14_-ISF-8 and the CPQ_11–14_-RSF-8, respectively. There was also no ceiling effect on any of the short-forms. The average level of impact identified by the 16-item questionnaires was almost identical, while on the 8-item questionnaires it differed by only one score point (Table 3).

**Table 3:** Descriptive statistics for the CPQ_11–14_-ISF:16, CPQ_11–14_-ISF:8, CPQ_11–14_-RSF:16 and CPQ_11–14_-RSF:8 scores

**Short-form:**	**Range of possible values**	**Mean (SD)**	**Range of scores**	**% with score of 0**	**% with max score**
**CPQ_**11–14**_-ISF:16**	0–64	13.8 (8.4)	1–40	0.0	0.0
**CPQ_**11–14**_-RSF:16**	0–64	13.6 (8.4)	1–37	0.0	0.0
**CPQ_**11–14**_-ISF:8**	0–32	7.4 (4.4)	0–24	0.8	0.0
**CPQ_**11–14**_-RSF:8**	0–32	6.6(4.6)	0–22	4.1	0.0

The CPQ_11–14_-ISF-16 and the CPQ_11–14_-RSF-16 found, respectively, 47.2% and 44.7% children who experienced 1 or more impacts 'Often' or 'Everyday/Almost everyday'. The CPQ_11–14_-ISF-8 was more sensitive in detecting these children than the CPQ_11–14_-RSF-8 (37.4% vs. 30.7%).

The scores standardized to a scale of 0 to 100 were on average higher on the short-form questionnaires than the CPQ_11–14_: 17.0 ± 11.4. Respectively, the mean values for the CPQ_11–14_-ISF-16, the CPQ_11–14_-RSF-16, the CPQ_11–14_-ISF-8 and the CPQ_11–14_-RSF-8 were 21.6 ± 13.2, 21.3 ± 13.2, 23.0 ± 13.8 and 20.7 ± 14.5. All differences were statistically significant (p < 0.001; paired T-test).

### Criterion validity

All short forms except the CPQ_11–14_-ISF-8 (rho = 0.87) were almost perfectly correlated with the long-form questionnaire (Table 4). While the correlation coefficients for the CPQ_11–14_-ISF-16 and the CPQ_11–14_-RSF-16 were nearly identical, the correlation coefficient for the CPQ_11–14_-ISF-8 was somewhat lower than the correlation coefficient for the CPQ_11–14_-RSF-8 (0.87 vs. 0.95) (Table 4).

**Table 4:** Criterion validity – rank correlations between scores of the short-forms and the long-form of CPQ_11–14 _(n = 123)

	**Long-form CPQ_**11–14**_**	
**Short-form:**	rho^a^	P^b^
**CPQ_**11–14**_-ISF:16**	0.96	0.000
**CPQ_**11–14**_-RSF:16**	0.98	0.000
**CPQ_**11–14**_-ISF:8**	0.87	0.000
**CPQ_**11–14**_-RSF:8**	0.95	0.000

^a ^Spearman's correlation coefficient; ^b ^p-value

### Construct validity

#### Discriminant construct validity

All short forms detected differences in impact on the quality of life among the three clinical groups in the expected direction. That is, the scores were highest in the oro-facial group, lower in the orthodontic group and lowest in the paediatric dentistry group (Table 5). The differences were statistically significant except on the CPQ_11–14_-RSF-16. The relative validity coefficients (RV) for the CPQ_11–14_-ISF-16, the CPQ_11–14_-RSF-16, the CPQ_11–14_-ISF-8 and the CPQ_11–14_-RSF-8 were 1.16, 0.85, 1.08 and 1.18, respectively. Therefore, the CPQ_11–14_-ISF-16, the CPQ_11–14_-ISF-8 and the CPQ_11–14_-RSF-8 demonstrated increased precision (8%-18%), while the precision of the CPQ_11–14_-RSF-16 was reduced by 15% compared with the original CPQ_11–14_.

**Table 5:** Discriminant construct validity – scores of the short forms of the CPQ_11–14 _by clinical group

	**Paedodontic **(n = 32)		**Orthodontic **(n = 52)		**Oro-facial **(n = 39)		**P**^**a**^
Short-form:	Median	Mean (SD)	Median	Mean (SD)	Median	Mean (SD)	
CPQ_11–14_-ISF:16	10.0	11.9 (9.4)	12.0	13.0 (7.6)	14.0	16.5 (8.3)	0.027
CPQ_11–14_-RSF:16	9.5	11.9 (9.2)	12.0	13.0 (7.4)	12.0	15.9 (8.8)	0.101
CPQ_11–14_-ISF:8	6.0	6.8 (5.5)	6.0	7.7 (3.7)	8.0	8.8 (4.2)	0.024
CPQ_11–14_-RSF:8	4.0	5.3 (4.6)	6.0	6.4 (4.1)	6.0	8.0 (5.1)	0.030

^a ^p-values obtained from Kruskal-Wallis test

Within the paedodontic group, on all short forms the mean score for children with 10 or more decayed tooth surfaces (n = 6) was higher than for children with fewer than 10 decayed tooth surfaces (n = 19). The differences were 2.5 (CPQ_11–14_-ISF-16), 2.2 (CPQ_11–14_-RSF-16), 1.3 (CPQ_11–14_-ISF-8) and 1.9 (CPQ_11–14_-RSF-8) score points. However, none of the differences was statistically significant. Within the orthodontic group, the mean scores were higher for children with Class II Division 1 (n = 21) than for children with Class I (n = 13). They were 16.5 vs. 10.8 for the CPQ_11–14_-ISF-16; 16.9 vs. 11.2 for the CPQ_11–14_-RSF-16; 8.1 vs. 5.7 for the CPQ_11–14_-ISF-8; and 8.1 vs. 5.3 for the CPQ_11–14_-RSF-8. All differences were statistically significant (p < 0.05; T-test). Within the oro-facial group, the mean scores for children with either isolated cleft of the lip or isolated cleft of the palate (n = 11) were higher compared to the mean scores for children with either unilateral or bilateral complete lip and palate cleft (n = 18). The score point differences were 4.4 on the CPQ_11–14_-ISF-16; 4.2 on the CPQ_11–14_-RSF-16; 0.7 on the CPQ_11–14_-ISF-8; and 2.6 on the CPQ_11–14_-RSF-8. The differences were not statistically significant.

#### Correlational construct validity

All short-form questionnaires demonstrated positive significant correlations with the ratings of oral health and overall well-being (Table 6). The rank correlation coefficients were consistently higher for the rating of overall well-being than the rating of oral health (Table 5). The strength of correlation was almost identical regardless of the method of development or the number of questions, as the coefficients ranged from 0.19 to 0.23 for the oral health rating and from 0.36 to 0.42 for the overall well-being rating amongst the four short-form questionnaires.

**Table 6:** Construct validity – correlations between short forms scores and oral health and overall well-being global ratings (n = 123)

	**Oral health**		**Overall well-being**	
**Short-form:**	**rho**^**a**^	**P**^**b**^	**rho**	**P**
**CPQ_**11–14**_-ISF:16**	0.21	0.020	0.40	0.000
**CPQ_**11–14**_-RSF:16**	0.20	0.026	0.42	0.000
**CPQ_**11–14**_-ISF:8**	0.19	0.033	0.39	0.000
**CPQ_**11–14**_-RSF:8**	0.23	0.010	0.36	0.000

^a ^Spearman's correlation coefficient; ^b ^p-value

### Reliability

Cronbach's alpha for the CPQ_11–14_-ISF-16, the CPQ_11–14_-ISF-8, the CPQ_11–14_-RSF-16 and the CPQ_11–14_-RSF-8 was 0.83, 0.83, 0.71 and 0.73, respectively. They indicate substantial internal consistency reliability for all short-form questionnaires. There was little change in the alphas when individual items were deleted. Corrected item total correlations were of the same magnitude for the four short forms. The ICCs ranged from 0.71 to 0.77 suggesting substantial test-retest reliability (Table 7) [20]. All short forms demonstrated substantial to high internal consistency and substantial test-retest reliability for each of the the clinical groups studied (Table 8).

**Table 7:** Reliability Statistics: Short Forms of the CPQ_11–14_

**Short-form:**	**Cronbach's α (n = 123)**	**Range of α's if items deleted**	**Range of corrected item total correlations**	**ICC^**a **^(n = 65)**
**CPQ_**11–14**_-ISF:16**	0.83	0.81–0.83	0.30–0.57	0.77
**CPQ_**11–14**_-RSF:16**	0.83	0.81–0.83	0.37–0.59	0.75
**CPQ_**11–14**_-ISF:8**	0.71	0.67–0.70	0.31–0.47	0.73
**CPQ_**11–14**_-RSF:8**	0.73	0.69–0.72	0.30–0.53	0.71

^a ^Intraclass correlation coefficient

**Table 8:** Reliability Statistics: Short Forms of the CPQ_11–14 _by Clinical Group

	**Cronbach's alpha**				**Intraclass correlation coefficient**^**a**^			
Short-form CPQ_11–14_:	ISF:16	RSF:16	ISF:8	RSF:8	ISF:16	RSF:16	ISF:8	RSF:8
Paedodontic group	0.88	0.87	0.85	0.73	0.77	0.75	0.79	0.70
Orthodontic group	0.81	0.79	0.61	0.66	0.69	0.70	0.67	0.65
Oro-facial group	0.80	0.84	0.62	0.77	0.74	0.80	0.70	0.74

^a ^Obtained from one-way random effect parallel model

## Discussion

In this study, short forms of the Child Perceptions Questionnaire for 11–14-year-olds (CPQ_11–14_) have been developed, tested for cross-sectional validity and reliability, and compared with the original instrument in terms of measurement sensitivity and discriminative properties. Each of the shortening techniques that were used, the item impact method and the stepwise regression, produced a 16-item and an 8-item measure. Measures of different lengths were developed to facilitate the administration of the questionnaire in clinical settings (16-item short-form) and in epidemiological surveys involving general populations (8-item short-form). To preserve the multidimensionality of the instrument so that it continues to conform to the WHO definition of health and the contemporary conceptualization of child health, the questions were selected from all domains in the CPQ_11–14_. Each domain contributed four questions for the 16-item short-forms and two questions for the 8-item short-forms. Previous research has indicated that versions of short-form questionnaires generated by the two approaches we used often differ in their content and measurement properties. The 16-item short forms generated in this study, i.e. CPQ_11–14_-ISF-16 and the CPQ_11–14_-RSF-16, had 14 questions in common (Table 1). The questions specific to these two questionnaires concern functional limitations and social well-being. On the contrary, the 8-item versions shared only 2 questions (Table 2). However, this difference in content had little effect on the performance of the two versions, reflecting the fact that Cronbach's alphas in each domain in the long form of the CPQ_11–14 _were high.

The questionnaires demonstrated considerable measurement sensitivity as the range of the scores showed that the short forms are detecting substantial variability in children's perceptions of their OHRQoL. The 16-item measures did not show floor-effects, while they were minimal for the 8-item questionnaires: 0.8% (CPQ_11–14_-ISF-8) and 4.1% (CPQ_11–14_-RSF-8). On average, all short forms detected higher levels of impact on the quality of life than the CPQ_11–14_. This can be explained by the fact that the questions selected for the short forms concern problems that children reported as the most frequent and the most bothersome. The lower scoring questions that were deleted when generating the short forms contribute to the CPQ_11–14 _scores and, consequently, lower the values of its standardized score.

The high correlations between the CPQ_11–14 _and the short-forms suggest that they are measuring the same construct. The association was somewhat stronger for the regression short-forms in comparison to impact short-forms, which can be explained by the fact the questions selected for the regression short-forms are those that explain the most variation in the overall scores of the CPQ_11–14_.

Reducing the number of questions in a questionnaire inevitably affects its content validity. Although content relevance remains intact, content coverage (i.e. the extent to which the questionnaire represents the construct of interest) is diminished. This, in turn, has the potential to compromise a measure's construct validity. Furthermore, since the reliability of a measure is a function of its length, the reduced number of questions may further attenuate construct validity by increasing the measurement error. However, the findings presented in this paper indicated that all short-forms have good construct validity since they were positively correlated with both global ratings. The correlation coefficients, as predicted, were lower for the rating of oral health than the rating of well-being. They were also either identical or very similar to the correlation coefficients found for the long form of the CPQ_11–14 _(0.23 and 0.40 for these two global ratings, respectively).

The construct validity of the short forms is further supported by the results of testing their ability to detect the hypothesized gradient in the impact of paedodontic, orthodontic and oro-facial conditions on children's quality of life. Although the score differences found on the CPQ_11–14_-RSF-16 were not statistically significant, they were in the expected direction and similar to the differences found on the CPQ_11–14_-ISF-16. The RV coefficients indicated that the statistical precision of the short forms in this study was similar to the statistical precision of the CPQ_11–14_, since all had values close to one. Gradients were also observed within the three clinical groups according to the severity of the condition. However, because clinical data were not available for some children, sample sizes were small and the differences mostly non-significant.

Although the reliability coefficients for the short forms were lower than those estimated for the CPQ_11–14 _(Cronbach's α = 0.91; ICC = 0.90), they all exceed standards for group-level comparisons [6,21]. However, they suggest possible limitations of the short forms for smaller-scale cross-sectional studies, especially when the samples involved show low variations in their OHRQoL. The same holds for individual-level assessments since they require that reliability coefficients are at least 0.90 [6,21].

A weakness of this study is that none of the short forms was administered on its own. Instead, the data collected in the validation study for the original questionnaire were used to evaluate their measurement properties. The possibility is that children may have responded differently had the short forms been the data collection instruments. However, it seems reasonable to assume that this is not very likely as Schofield et al. [22] found no significant differences in the mean summary scores when the SF-12 was embedded in the SF-36 as opposed to when it was administered by itself to an equivalent independent sample.

The study provides evidence about measurement sensitivity and discriminative properties (i.e. construct validity and reliability) of the 16-item and 8-item short forms of the Child Perceptions Questionnaire for 11–14-year-old children developed using the item impact method and stepwise regression. However, these are preliminary findings based on convenience sampling of a clinical population and further testing in replicated studies involving clinical and general samples of children in various settings is necessary. If the cross-sectional properties of the short forms are confirmed then, since they perform equally well but vary in their content, the one that is selected for a study would depend on the purpose of the investigation, the population studied and research context. This is of a particular importance with respect to the 8-item versions as they share only two questions. Moreover, if an 8-item version is used analysis of overall scale scores is possible but not analysis at the level of the individual domains. The number of items per domain is insufficient for this purpose.

A final consideration is whether the item impact or regression approach is better when developing a short form measure. From a statistical point of view the latter may be contraindicated because the distribution of the data derived from a quality of life questionnaire will, more likely than not, violate the assumptions of linear regression analysis. Moreover, the use of forward stepwise regression in this context may be compromised by the part-whole correlation effect (10) since it often results in the wrong variables being selected. Because of these problems Coste et al (10) suggest that an expert-based approach if preferable. While these statistical considerations are important, the study reported here suggests that, in practice, the regression approach performs reasonably well. The advantage of the item impact approach is that it selects those items of most importance to the people who will be completing the questionnaire who may be considered to be the ultimate experts concerning the impact of a given condition on the quality of life (11). Juniper et al (11) suggests that the choice of approach is largely a philosophical matter in which an investigator must decide whether patients' views or statistical considerations are of most importance. Locker and Allen (15) take the view that the method of developing a short form questionnaire is less important than its content and properties, a view that is supported by the results of this study. However, since different approaches can result in different short form instruments which may vary in their items and their properties, investigators shortening a measure should consider using more than one approach to determine the effect of method on outcome.

## Authors' contributions

AJ and DL conceived of the study and with GG were responsible for the study design. GG had previously developed the item impact approach for constructing health-related quality of life questionnaires and for producing short forms. AJ coordinated the study, undertook the statistical analysis and drafted the manuscript. DL assisted in drafting the manuscript and was responsible for the revised version. All authors read and approved the final manuscript.
